# Molecular pathways associated with blood pressure and hexadecanedioate levels

**DOI:** 10.1371/journal.pone.0175479

**Published:** 2017-04-12

**Authors:** Cristina Menni, Sarah J. Metrustry, Georg Ehret, Anna F. Dominiczak, Phil Chowienczyk, Tim D. Spector, Sandosh Padmanabhan, Ana M. Valdes

**Affiliations:** 1 Department of Twin Research and Genetic Epidemiology, Kings College London, London, United Kingdom; 2 Cardiology, Geneva University Hospital, Geneva, Switzerland; 3 Institute of Cardiovascular and Medical Sciences, College of Medical, Veterinary and Life Sciences, University of Glasgow, Glasgow, United Kingdom; 4 Department of Clinical Pharmacology, King's College London, London, United Kingdom; 5 Academic Rheumatology Clinical Sciences Building, Nottingham City Hospital, Nottingham, United Kingdom; George Washington University School of Medicine and Health Sciences, UNITED STATES

## Abstract

The dicarboxylic acid hexadecanedioate is associated with increased blood pressure (BP) and mortality in humans and feeding it to rats raises BP. Here we aim to characterise the molecular pathways that influence levels of hexadecanedioate linked to BP regulation, using genetic and transcriptomic studies. The top associations for hexadecanedioate in a genome-wide association scan (GWAS) conducted on 6447 individuals from the TwinsUK and KORA cohorts were tested for association with BP and hypertension in the International Consortium for BP and in a GWAS of BP extremes. Transcriptomic analyses correlating hexadecanedioate with gene expression levels in adipose tissue in 740 TwinsUK participants were further performed. GWAS showed 242 SNPs mapping to two independent loci achieving genome-wide significance. In rs414056 in the *SCLO1B1* gene (Beta(SE) = -0.088(0.006)*P* = 1.65 x 10^−51^, *P* < 1 x 10^−51^*)*, the allele previously associated with increased risk of statin associated myopathy is associated with higher hexadecanedioate levels. However this SNP did not show association with BP or hypertension. The top SNP in the second locus rs6663731 mapped to the intronic region of *CYP4Z2P* on chromosome 1 (0.045(0.007), *P* = 5.49x10^-11^). Hexadecanedioate levels also correlate with adipose tissue gene-expression of the 3 out of 4 *CYP4* probes (*P*<0.05) and of alcohol dehydrogenase probes (Beta(SE) = 0.12(0.02); *P *= 6.04x10^-11^). High circulating levels of hexadecanedioate determine a significant effect of alcohol intake on BP (SBP: 1.12(0.34), P = 0.001; DBP: 0.70(0.22), P = 0.002), while no effect is seen in the lower hexadecanedioate level group. In conclusion, levels in fat of *ADH1A*, *ADH1B* and *CYP4* encoding enzymes in the omega oxidation pathway, are correlated with hexadecanedioate levels. Hexadecanedioate appears to regulate the effect of alcohol on BP.

## Introduction

Hypertension represents a major global disease burden, but discovering pathways for blood pressure (BP) regulation has been challenging. A number of recent studies by our group and others have found several metabolites to be correlated with BP [1–4]. Circulating levels of the dicarboxylic fatty acid hexadecanedioate are associated with increased BP in three independent cohorts and are linked to increased risk of mortality. Evidence for a causal role was obtained by feeding this compound to rats resulting in significant increases in BP, indicating that it is not a by-product, but a cause of high BP [2]. In addition, a recent study has shown a significant effect of hexadecanedioate on incident heart failure which appeared to be causal [5]. Hexadecanedioate is a by-product of omega oxidation of fatty acids, a minor pathway for fatty acid oxidation used when beta oxidation is deficient. The second step is carried out by the enzyme alcohol dehydrogenase. However, the underlying determinants of its variation are still unknown.

We hypothesized that identifying the genetic contribution to circulating levels of hexadecanedioate and genes whose expression is highly correlated to this compound should reveal some of the pathways defining the regulation and pathology of how hexadecanedioate affects BP.

## Methods

The study participants were twins enrolled in the TwinsUK Registry, a national register of adult twins recruited via media campaigns without selecting for any particular disease and phenotype [6].

### Genome-wide association

Here we dissected the hexadecanedioate genome-wide association scan (GWAS) data that was previously generated and published as part of our GWAS-metabolomics study[7]. Briefly, non targeted mass spec metabolomic profiling and quantification was conducted on fasting serum and plasma samples by the metabolomics provider Metabolon, Inc. (Durham, NC, USA) [8]. GWAS (in the HapMap 2–based imputed genotype data set) was conducted on 6056 individuals from TwinsUK and 1768 from KORA as previously described [7]. Association results were combined in Metal[9] using inverse variance meta-analysis based on effect size estimates and standard errors, adjusting for genomic control.

We tested the multiple single nucleotide polymorphisms (SNPs) associated with hexadecanedioate for association with BP in the International Consortium for Blood Pressure (ICBP)[10] and with hypertension in the BP-extreme GWAS [11] study. Briefly, the ICBP consortium is an international effort to investigate BP genetics. The consortium was formed by two parent consortia, the CHARGE-BP consortium (Cohorts for Heart and Aging Research in Genomic Epidemiology—blood pressure) and the GBPGEN consortium (Global Blood Pressure Genetics Consortium). The BP-extreme GWAS consists of 1621 hypertensive cases and 1699 controls from respectively the top 2% and the lower 9.2% of the BP distribution of the Swedish population [11].

### Gene expression

The association of hexadecanedioate with gene-expression levels in fat and lymphoblastoid cell line (LCL) was tested in 740 females from the TwinsUK cohort using random intercept linear regression after adjusting for age, BMI, metabolite batch, expression batch and family relatedness. Gene expression was analysed with the Illumina Human HT-12 V310 as previously reported[12].

### Post-genomic functional analysis

Post-genomic functional analysis was undertaken using the Database for Annotation, Visualization and Integrated Discovery (DAVID) [13]. This is an online tool to which a list of genes can be submitted and subsequently results are generated regarding the genes’ involvement in biological processes [13]. The gene list was comprised of genes corresponding to all SNPs with a P value of p<0.0001 in the GWAS analysis. The BioCarta and Kegg pathways maps were used for functional annotation.

### Pathway analysis

Pathway analysis was carried out on the microarray results using a list of genes corresponding to probes with p values less than P<0.00002 (n = 52).

### Association with alcohol intake

We assessed the association between alcohol intake (measured via Food Frequency Questionnaire) and BP stratifying by hexadecanedioate levels by using random linear regression adjusting for age, age^2^, BMI and family relatedness.

The study was approved by St. Thomas' Hospital Research Ethics Committee. All participants provided informed written consent.

TwinsUK metabolomics, and phenotypic data are publicly available upon request on the department website (http://www.twinsuk.ac.uk/data-access/accessmanagement/).

## Results

**Fig 1:**
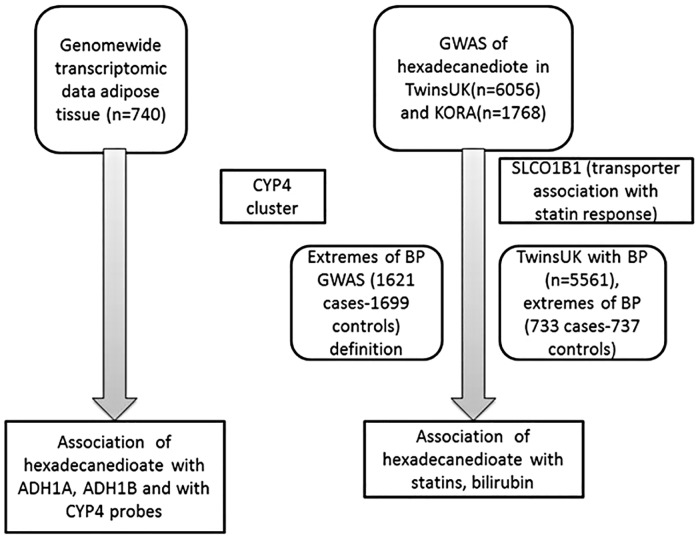
Flowchart of the study design.

The flowchart of the study design is depicted in Fig 1.

### Genetics

**Fig 2:**
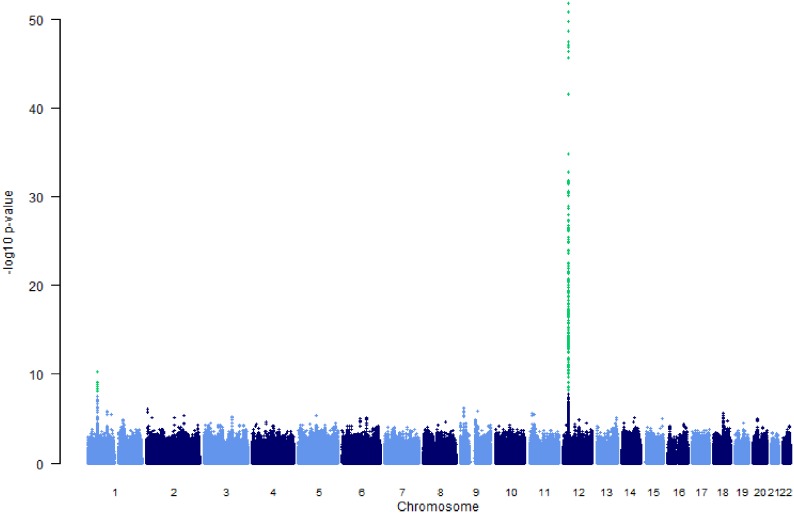
Manhattan plot showing genome-wide P values from association analysis of hexadecanedioate in the TwinsUK-KORA meta-analysis. The y axis shows the −log10 P values of SNPs, and the x axis shows their chromosomal positions.

**Fig 3:**
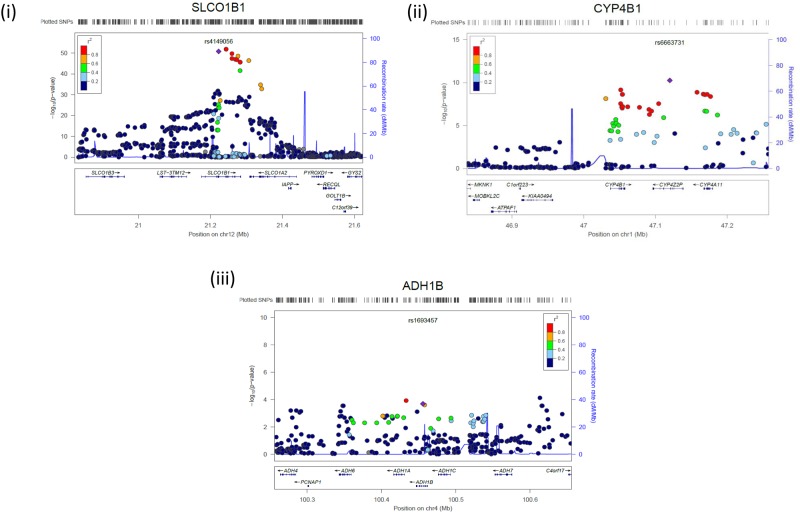
Association plot of the genomic region around (i) SLCO1B1, (ii) CYP4B1 and (iii)ADH1B showing both type and imputed SNPs with location of genes and recombination rate. *P* values of SNPs are plotted (as −log_10_ *P*) against their physical position on the chromosomes (NCBI Build 36). Estimated recombination rates from the HapMap CEU population show the local LD structure. The color of each SNP indicates linkage disequilibrium with the index SNP (rs4149056 or rs6663731 or rs1693457 respectively) based on pairwise *r*^2^ values from HapMap CEU data.

The results of the GWAS study from our previously published metabolomics GWAS [7]are presented in Fig 2. 242 SNPs are associated with circulating levels of hexadecanedioate achieving genome-wide significance (*P*<5x10^-8^)[7]. All these SNPs map to two genes: the Solute Carrier Organic Anion Transporter Family, Member 1B1 (*SLCO1B1)* on chromosome 12 and a cytochrome 4 cluster on chromosome 1 which contains the genes *CYP4A11*, *CYP4B1 and CYP4Z2P*. The locus plots of *SLCO1B1* and the CYP4 cluster are presented in Fig 3(i) and 3(ii) respectively.

Among the top SNPs, rs4149056 is a non-synonymous polymorphism on the *SLCO1B1* gene on chromosome 12 (Beta(SE) = -0.088(0.006)*P* = 1.65 x 10^−51^), encoding an Val174Ala amino acid change. This SNP did not show association with BP[10] or hypertension (HTN)[11]. The hexadecanedioate increasing allele of this SNP has previously been associated with statin related myopathy [14], so we further tested whether concomitant use of statins was associated with different levels of hexadecanedioate. We find that use of statins correlates with significantly lower levels of hexadecanedioate (-0.157(0.07), *P *= 0.0347) which remains essentially unchanged if adjusted for SBP (-0.156(0.07), *P* = 0.0319).

SNP rs11045656 on *SLCO1B1* showed nominal association with HTN (Beta(SE) = 0.06(0.03), P = 0.02) in the GWAS of BP extremes (Table 1)[11].

**Table 1:** Association between SNPs in the *SLCO1B1* gene and *CYP4* cluster and hexadecanedioate levels in the meta analysis carried out by Shin et al (2014)[7], in the extremes of BP carried out by Padmanabhan et al (2010)[11], in the ICBP consortium GWAS(2011)[10] and with risk of statin associated myopathy as reported (SEARCH collaborative group 2008)[14].

Gene	SNP	EA	hexadecanedioate-TUK KORA meta-analysis Nat Gen			HTN extreme GWAS			ICBP			Risk of statin associated myopathy		
			BETA	SE	P	BETA	SE	P	BETA	SE	P	BETA	SE	P
SLCO1B1	**rs4149056**	**T**	-0.088	0.006	**1.65x10**^**-51**^	**-0.006**	**0.031**	**0.86**	**-0.12**	**0.13**	0.34*	**-1.51**	**0.258**	**4.0x10**^**-9**^
	rs2900478	A	0.087	0.006	2.02x10^-50^	0.022	0.029	0.50						
	rs7969341	A	-0.086	0.006	2.67x10^-49^	-0.022	0.029	0.50						
	**rs4363657**	**T**	-0.085	0.006	**3.92x10**^**-48**^	**-0.022**	**0.029**	**0.49**				**-1.51**	**0.258**	**4.0x10**^**-9**^
	rs4149081	A	0.084	0.006	9.67x10^-48^	0.021	0.029	0.50						
	rs11045879	T	-0.084	0.006	1.56x10^-47^	-0.022	0.029	0.49						
	rs2199680	A	-0.084	0.006	4.52x10^-47^	-0.018	0.029	0.57						
	rs12366582	A	-0.083	0.006	2.28x10^-46^	-0.019	0.029	0.55						
	rs12369881	A	0.083	0.006	2.28x10^-46^	0.019	0.029	0.54						
	rs12371604	T	-0.069	0.005	2.85x10^-42^	-0.002	0.026	0.96						
	rs4149054	A	0.054	0.005	6.26x10^-26^	0.027	0.027	0.37						
	rs4149050	T	-0.052	0.005	1.23x10^-24^	-0.027	0.027	0.37						
	rs4149058	A	-0.052	0.005	2.06x10^-24^	-0.026	0.027	0.37						
	rs4149057	T	0.039	0.004	2.00x10^-19^	0.023	0.023	0.36						
	**rs11045656**	**A**	0.026	0.004	**5.39x10**^**-9**^	**0.064**	**0.025**	**0.02**						
CYP4	rs6663731	A	0.045	0.007	5.49x10^-11^	0.067	0.035	0.08	-0.041	0.147	0.92			
	rs9332998	T	0.04	0.007	4.19x10^-9^	0.068	0.036	0.08	-0.002	0.148	0.99			

* Beta(SE), P Value for SNP rs12317268 that is in complete LD (R2 = 1) with rs4149056

A SNP in *SLCO1B1*, monomorphic in Caucasians, has also been implicated in both levels of hexadecanedioate and risk heart failure in African Americans [5].

The top SNP in the second locus rs6663731 mapped to the intronic region of *CYP4Z2P* on chromosome 1 (0.045(0.007), *P* = 5.49x10^-11^). No association with HTN was detected for the SNPs mapping to the cytochrome 4 cluster. However, circulating levels of hexadecanedioate correlate with adipose tissue gene-expression levels of probes mapping to *CYP4B1* and *CYP4Z2P* (S1 Table). No probes mapping to *CYP4A11* passed quality control in our gene expression data.

### Gene expression

Gene expression in 740 abdominal fat and LCL samples from the TwinsUK cohort were tested for association with hexadecanedioate levels. 23 gene transcripts showed significant association with hexadecanedioate levels in fat after adjusting for multiple testing (Bonferroni P = 0.05/[23644 probes x 2 tissues] = 1x10^-6^). The top association is with the Alcohol Dehydrogenase 1B (Class I), Beta Polypeptide (*ADH1B)* gene on chromosome 4. SNPs on the gene are also associated with circulating hexadecanedioate levels in our metabolomics GWAS [7]. No significant associations were observed in LCL tissue. The significant expression results from the analysis of fat samples are presented in Table 2.

**Table 2:** Significant expression results for hexadecanedioate in adipocytes.

Gene	Probe	Beta	SE	P
ADH1B	ilmn_1811598	0.12	0.02	6.04x10^-11^
GSDMB	ilmn_1666206	0.12	0.02	5.55x10^-10^
CIDEA	ilmn_1788184	0.14	0.02	8.01x10^-10^
CIDEA	ilmn_2390318	0.13	0.02	1.03x10^-9^
MOCS1	ilmn_1798624	0.06	0.01	4.25x10^-9^
GSDMB	ilmn_2347193	0.10	0.02	5.02x10^-9^
CEACAM1	ilmn_1716815	0.05	0.01	8.71x10^-9^
SFRP2	ilmn_1722898	-0.14	0.02	1.03x10^-8^
FAM184A	ilmn_1696699	0.05	0.01	1.57x10^-8^
RGS17	ilmn_1725485	0.07	0.01	1.65x10^-8^
SLC19A3	ilmn_1716359	0.09	0.02	1.91x10^-8^
MAGED1	ilmn_1775522	-0.05	0.01	3.12x10^-8^
HDDC3	ilmn_1781638	0.05	0.01	3.27x10^-8^
KHDRBS3	ilmn_1691747	0.05	0.01	3.79x10^-8^
GLYCTK	ilmn_1791222	0.09	0.02	7.24x10^-8^
PQLC1	ilmn_1798620	0.04	0.01	8.88x10^-8^
EHBP1	ilmn_1803348	-0.06	0.01	1.07x10^-7^
ANG	ilmn_1760727	0.06	0.01	2.91x10^-7^
MT1M	ilmn_1657435	0.06	0.01	5.23x10^-7^
SPSB1	ilmn_1714170	-0.07	0.01	5.66x10^-7^
NXNL1	ilmn_1742917	0.06	0.01	5.71x10^-7^
ECHDC2	ilmn_1671568	0.05	0.01	7.56x10^-7^
GART	ilmn_1679476	0.03	0.01	8.80x10^-7^

### Association with alcohol intake

Because genetic variants at the *ADH1B* gene have been implicated in alcohol induced HTN [15], we explored the association of alcohol and hexadecanedioate on BP in the TwinsUK dataset. We first assessed whether alcohol intake influenced the effect of hexadecanedioate on BP. After adjusting for alcohol consumption (in a log scale) age, age^2^, BMI, family relationship, we find that circulating levels of hexadecanedioate are significantly associated with both SBP (Beta(SE) = 1.30(0.29),P = 1.1x 10^−5^) and DBP (0.74(0.19), P = 7.5x 10^−5^) supporting our previous findings. Overall alcohol consumption is significantly associated with SBP and DBP after adjusting for covariates (SBP:0.54(0.19), P = 6.2x10^-3^) and DBP (0.49(0.13, P = 2.6x10^-4^). However when stratifying for hexadecanededioate levels, the association between alcohol and BP is present in the individuals in the top tertiles of hexadecanedioate circulating levels (SBP: 1.12(0.34), P = 0.001; DBP: 0.70(0.22), P = 0.002). On the other hand the level of alcohol intake has no influence on the association between hexadecanedioate levels and BP in those with low hexadecanedioate levels (Table 3). When we stratified the cohort into participants who drink 1 standard alcohol drink per day or less (14 g per day) and those who drink more we find that the effect of hexadecanedioate on blood pressure is the same regardless of alcohol intake (low alcohol: 1.30(0.32, P = 4.5x10^-5^; high alcohol: 1.30(0.36), P = 3.2x10^-4^).

**Table 3:** Alcohol intake and BP stratified by hexadecanedioate levels adjusting for age, age^2^, BMI and family relatedness.

Hexadecanedioate	SBP		DBP	
	Beta(SE)	P	Beta(SE)	P
**Overall**	0.54(0.19)	6.2x10^-3^	0.49(0.13)	2.6x10^-4^
**Low tertile**	0.06(0.35)	0.86	0.21(0.21)	0.84
**Middle tertile**	0.26(0.33)	0.44	0.45(0.23)	0.05
**High tertile**	1.12(0.34)	0.001	0.70(0.22)	0.002

This result suggests that high levels of hexadecanedioate may be indicating a dysfunction or a saturation of enzymatic pathways within the liver related to alcohol metabolism.

### Pathway analysis

The results of this analysis in the TwinsUK dataset(see S2 Table shows some functional clustering of genes, particularly relating to cytoskeleton organisation, cell motility, migration and projection as well as regulation of apoptosis all of which have been involved in endothelial dysfunction. However after applying a Benjamini correction, for multiple tests none of these *P* values remained significant (see S2 Table).

## Discussion

In this study we investigated some of the molecular pathways underlying the fatty acid hexadecanedioate. Exploting our previously published metabolomics GWAS[7], we identified two loci, mapping to *SLCO1B1* and to the cluster encoding *CYP4* genes strongly associated to circulating levels of hexadecanedioate with 242 SNPs passing Bonferroni correction for multiple testing.

Our gene-expression analyses show that circulating levels of hexadecanedioate are nominally correlated with adipose tissue levels of probes mapping to *CYP4B1* and *CYP4Z2P*. The fact that CYP cluster genes correlate with hexadecanedioate but do not reach significance for over-expression may suggest that such over-expression may be occurring in other (not investigated) tissues in which their functions regarding blood pressure are more important (e.g., vessels or kidney). The alcohol dehydrogenase 1B (class I), beta polypeptide (ADH1B) is however the strongest gene whose expression is associated with hexadecenedioate after adjusting for multiple testing. Although the effect of hexadecanedioate on BP remains unvaried after adjustment for alcohol intake [2] these data suggest that there may be an important interaction between alcohol intake and hexadecandenedioate levels with regards to their effect on BP.

### SLCO1B1

The strongest genetic association seen with hexadecanedioate maps to *SLCO1B1*, an association previously reported [16, 17] in a metabolome-wide genetic study in Caucasians[17] and also in African Americans [5].

The *SLCO1B1* gene encodes OATP1B1, (also named OATP2, OATP-C and LST-1) which is mainly expressed on the sinusoidal membrane of human hepatocytes. Substrates of OATP1B1 include endogenous organic anions such bilirubin, estradiol, prostaglandin 2, leukotrienes C4 and thyroxine, and structurally diverse drugs, such as statins, antibiotics (Rifampicin) antivirals (Saquinavir) and some anti-hypertensive drugs (valsartan)[18].

The functional variants that causes reduced function of OATP1B1 and identified as increasing the risk of statin myopathy (rs414056 and rs4363657 and variants in linkage disequilibrium with them) are the most strongly associated with higher levels of hexadecanedioate.

We find no convincing evidence of an association between variants in *SLCO1B1* with HTN or BP in the two GWAS that we tested [10, 11].This lack of association is not due to lack of power: the ICBP GWAS is sufficiently powered to detect effects as those expected (0.4 mm Hg per allele) yet we failed to see a significant association with the hexadecanedioate associated SNPs in Caucasians. The effect of the variant allele at rs4149056 is -0.087, the effect of each SD of hexadecanedioate is 1.3 mm Hg per SD of hexadecanedioate. Therefore we expect an effect of 0.44 mmHg per allele difference in rs4149056 if hexadecanedioate is causative of blood pressure increase. A sample size of 42,672 individuals is needed to find this as statistically significant with p<0.05 with a MAF of 19% with 80% power, and 67161 for 94% power. The ICBP GWAS used a samples size of 69395 individuals and hence had over 94% power to detect the expected effect under the hypothesis that hexadecanedioate levels are causing an increase in blood pressure. One possible explanation for this finding is that it is intracellular hexadecanedioate levels that influence BP and not necessarily circulating levels The association with *SLCOB1* reflects circulating levels in large part determined by hepatic uptake of the compound but this association may not be related to intracellular levels of hexadecanedioate.

Hexadecanedioate is a product of omega-oxidation, a secondary fatty acid oxidation pathway. In the first step, an hydroxyl group is introduced onto the omega carbon. This reaction is carried out by certain members of the *CYP4* subfamilies or by two other *CYP450* enzymes, and the electron donor *NADPH*. The next step is the oxidation of the hydroxyl group to an aldehyde by NAD+ and is catalysed by alcohol dehydrogenase, whose subunits are encoded by genes *ADH1A*, *ADH1B* nad *ADH1C*[19]. The third step is the oxidation of the aldehyde group to a carboxylic acid by NAD+. The product of this step is a fatty acid with a carboxyl group at each end, i.e. a dicarboxylic fatty acid, such as hexadecanedioate[20].

### CYP4A11 / CYP4B1/ CYP4Z2P

The second strongest genes to be statistically associated with hexadecanedioate are the Cytochromes *CYP4A11*, *CYP4B1* and *CYP4Z2P* on chromosome 1 as shown in Fig 2(ii).

*CYP4B1* has been shown to be involved in prostaglandin metabolism[21] through the production of 12-hydroxyeicosatrienoic acid (12-HETE), a potent inflammatory and angiogenic eicosanoid.

**I**n rodent models, decreased expression of *CYP4A* results in increased epithelial sodium channel (ENaC) activity and salt-sensitive hypertension.

Increased CYP4Z2P- along with the functional CYP4Z1-3'UTR expression has been shown to promote tumor angiogenesis in breast cancer partly via miRNA-dependent activation of PI3K/Akt and ERK1/2[22]. This is relevant to BP regulation as apoptosis of endothelial cells is involved in endothelial dysfunction and the resulting vascular disease. In addition, the mRNA of the CYP4Z2P pseudogene has been shown to be expressed in tissues that play a role in cardiovascular regulation such as brain, heart arteries, kidney and adrenals[23]. It is possible that genetic variation at the CYPZ2P gene may be influencing apoptosis of endothelial cells or other cells via MAP kinases and PI3K. Hence, there may be a link between endothelial dysfunction and hexadecanedioate levels and the regulation of the two may be linked via the CYP4 encoded molecules or actions.

### ADH1B

The gene whose expression is most strongly associated with regards to hexadecanedioate is *ADH1B*(0.12(0.02), P = 6.04x10^-11^). In addition also gene expression levels of *ADH1A* are associated with hexadecanedioate levels (0.09(0.02), P = 1.19x10^-6^). These genes encode the second enzyme in the omega oxidation making this association logical. It further suggests that study of the relationships between alcohol, hypertension and hexadecanedioate is needed. We also find significant associations between circulating levels of hexadecanedioate and 3 SNPs on the ADH1B gene (P<0.0005) as shown in Fig 3(iii). This suggests that *ADH1B* is implicated in hexadecanedioate regulation. Though we find no association between SNPs on ADH1B in hypertension and no association has been reported in Caucasians, a role for *ADH1B* in hypertension has been found in Japanese men[15]. Here we report that hexadecanedioate appears to influence the role of alcohol on BP, but the opposite is not true and the association of hexadecanedioate is independent of alcohol intake.

Both cross-sectional and prospective epidemiological studies have established a relationship between hypertension and alcohol consumption [24, 25]. Excessive alcohol use can increase BP and cause antihypertensive drug resistance in a dose-dependent manner [26]. The mechanism(s) by which ethanol consumption leads to elevations in blood pressure is uncertain. However, the available data in humans are not sufficient to allow substantive conclusions [27]. Limitation of daily ethanol intake to no more than 1 ounce (30 mL) of 40% ethanol for most men and 0.5 ounces for women and smaller men results in little blood pressure effect [27]. In some cases, BP control is extremely difficult without total abstinence.

Our finding that alcohol intake has a much stronger effect in individuals with high hexadecanedioate may have therapeutic implications for the treatment of alcohol-induced hypertension suggesting that the strategies for reducing blood pressure may be different depending on the subject’s hexadecanedioate levels.

In conclusion, all three genes identified in this study as strongly associated with levels of hexadecanedioate have been previously linked to hypertension, but this association is not strong, or not present in Caucasians. Some of the effects previously reported (in other ethnic groups or in small studies) between *ADH1B*, *SLCOB1* and *CYPB4A11* and BP may be due to their link to hexadecanedioate. *SLCOB1* appears not to be associated with BP in spite of its very strong association with hexadecanedioate levels. Our data lend support to the use of intermediate phenotypes, in this case, a metabolite that contributes to BP regulation, to understand some of the pathways involved in BP regulation and cardiovascular risk.

## Supporting information

S1 Table Association of circulating levels of hexadecanedioate and CYP4 in adipocytes. (DOCX)

S2 Table Over-represented pathways for gene expression in adipose tissue correlated with circulating hexadecanedioate levels. (DOCX)
