# Supplementary material for: Molecular pathways associated with blood pressure and hexadecanedioate levels
Source: PLoS One. 2017 Apr 12;12(4):e0175479. doi: 10.1371/journal.pone.0175479 (PMC5389832; doi:10.1371/journal.pone.0175479)
Supplement: S1 Table — (DOCX) [file pone.0175479.s001.docx]

**S1 Table. Association of circulating levels of hexadecanedioate and CYP4 in adipocytes.**

| **Gene** | **Probe** | **Beta** | **SE** | **P** |
| --- | --- | --- | --- | --- |
| CYP4B1 | ilmn_1659215 | 0.04 | 0.01 | 0.001 |
| CYP4B1 | ilmn_2173291 | 0.03 | 0.01 | 0.015 |
| CYP4Z2P | ilmn_1702829 | 0.01 | 0.004 | 0.009 |
