# Supplementary material for: Molecular pathways associated with blood pressure and hexadecanedioate levels
Source: PLoS One. 2017 Apr 12;12(4):e0175479. doi: 10.1371/journal.pone.0175479 (PMC5389832; doi:10.1371/journal.pone.0175479)
Supplement: S2 Table — (DOCX) [file pone.0175479.s002.docx]

**S2 Table. Over-represented pathways for gene expression in adipose tissue correlated with circulating hexadecanedioate levels.**

| **Pathway** | **n genes** | **%** | **P-Value** | **Benjamini** |
| --- | --- | --- | --- | --- |
| actin cytoskeleton organization | 5 | 1.1 | 0.0029 | 0.89 |
| actin filament-based process | 5 | 1.1 | 0.0037 | 0.75 |
| cell motion | 6 | 1.4 | 0.008 | 0.87 |
| cell projection | 7 | 1.6 | 0.0093 | 0.65 |
| Pyrrolidone carboxylic acid | 3 | 0.7 | 0.011 | 0.77 |
| cytoplasm | 15 | 3.4 | 0.017 | 0.69 |
| active site:Proton acceptor | 6 | 1.4 | 0.019 | 0.98 |
| negative regulation of signal transduction | 4 | 0.9 | 0.021 | 0.98 |
| SH3 domain binding | 3 | 0.7 | 0.022 | 0.94 |
| positive regulation of apoptosis | 5 | 1.1 | 0.027 | 0.98 |
| positive regulation of programmed cell death | 5 | 1.1 | 0.027 | 0.97 |
| positive regulation of cell death | 5 | 1.1 | 0.028 | 0.95 |
| cytoskeleton organization | 5 | 1.1 | 0.028 | 0.93 |
| negative regulation of cell communication | 4 | 0.9 | 0.028 | 0.91 |
| amine binding | 3 | 0.7 | 0.028 | 0.84 |
| PIRSF001015:pyrimidine-specific ribonuclease/angiogenin | 2 | 0.5 | 0.034 | 0.59 |
| cell migration | 4 | 0.9 | 0.037 | 0.94 |
| positive regulation of developmental process | 4 | 0.9 | 0.037 | 0.92 |
| pancreatic ribonuclease activity | 2 | 0.5 | 0.038 | 0.81 |
| Pancreatic ribonuclease | 2 | 0.5 | 0.04 | 0.99 |
| RNAse_Pc | 2 | 0.5 | 0.042 | 0.72 |
| endoribonuclease activity, producing 3'-phosphomonoesters | 2 | 0.5 | 0.044 | 0.77 |
| calmodulin-dependent protein kinase activity | 2 | 0.5 | 0.047 | 0.71 |
| positive regulation of bone mineralization | 2 | 0.5 | 0.047 | 0.95 |
| localization of cell | 4 | 0.9 | 0.048 | 0.94 |
| cell motility | 4 | 0.9 | 0.048 | 0.94 |
| positive regulation of biomineral formation | 2 | 0.5 | 0.049 | 0.93 |
